# *So-Ochim-Tang-Gamibang*, a Traditional Herbal Formula, Ameliorates Depression by Regulating Hyperactive Glucocorticoid Signaling *In Vitro* and *In Vivo*

**DOI:** 10.1155/2020/8834556

**Published:** 2020-11-10

**Authors:** Mirim Jin, Sun Young Park, Hye Jin Choi, Younmin Shin, Eunho Chun, In Chul Jung, Jeong June Choi

**Affiliations:** ^1^Department of Microbiology, College of Medicine, Gachon University, Incheon 21999, Republic of Korea; ^2^Lee Gil Ya Cancer and Diabetes Institute, Gachon University, Incheon 21999, Republic of Korea; ^3^Department of Health Sciences and Technology, GAIHST Gachon University, Incheon 21999, Republic of Korea; ^4^Laboratory of Molecular Medicine, College of Korean Medicine, Daejeon University, Daejeon 34520, Republic of Korea; ^5^Department of Neuropsychiatry, Daejeon Korean Medicine Hospital of Daejeon University, Daejeon 35235, Republic of Korea

## Abstract

*So-ochim-tang-gamibang* (SOCG) is a Korean traditional medicine; it has previously been shown to be safe and effective against depression. Persistently increased levels of circulating glucocorticoids have been considered as a pathological mechanism for depression and associated with decreased neurotrophic factors in the hippocampus. This study investigated whether SOCG controls the hyperactivity of the hypothalamic-pituitary-adrenal (HPA) axis and the molecular mechanisms underlying its effects *in vivo* and *in vitro*. Wistar Kyoto (WKY) rats were subjected to restraint stress, where SOCG was orally administered to the animals for 2 weeks. An open field test (OFT), forced swimming test (FST), and sucrose preference test (SPT) were performed to explore the antidepressant activity of SOCG in WKY rats. Plasma levels of HPA axis hormones were measured by ELISA or western blotting analysis. The expression levels or activation of HPA axis-related signaling molecules such as brain-derived neurotrophic factor (BDNF), cAMP response element-binding protein (CREB), extracellular regulated kinase (ERK), and glucocorticoid receptors (GRs) in the brain were determined by real-time PCR and western blotting analysis. Furthermore, a corticosterone- (CORT-) induced cell injury model was established using SH-SY5Y cells to explore the antidepressive effects of SOCG *in vitro*. The results of the OFT, FST, and SPT revealed that SOCG ameliorated depressive-like behaviors in the WKY rats. The blood plasma levels of HPA axis hormones such as CORT, CORT-releasing hormone (CRH), and adrenocorticotrophic hormone were downregulated by SOCG. On the other hand, SOCG upregulated the phosphorylation of CREB and ERK in both the rat hippocampus and CORT-treated SH-SY5Y cells. Moreover, it also increased the GR expression. These results suggested that SOCG may improve depression by controlling hyperactive glucocorticoid signaling *via* the downregulation of HPA axis hormones and upregulation of GR.

## 1. Introduction

Depression is a highly prevalent psychiatric ailment characterized by repetitive events of sadness and a loss of interest in normal activities; the incidence of depression has been increasing worldwide. Although the pathogenesis of depression is complex and has not been understood completely, it has been accepted that excessive circulating levels of glucocorticoids (GCs) are associated with not only the onset of depression but also depression-induced deterioration of nerve regeneration [1]. In healthy subjects, acute stress induces the release of corticosterone- (CORT-) releasing hormone (CRH) from the hypothalamus, which subsequently induces the release of adrenocorticotrophic hormone (ACTH) from the pituitary gland. The ACTH reaches the adrenal gland, ultimately triggering the production of GCs against the stress [2]. The activity of the hypothalamic-pituitary-adrenal (HPA) axis is driven by glucocorticoid receptors (GRs), whose balanced expression level is thought to be an important factor in regulating the activity of the HPA axis. Particularly, in depression, a high concentration of circulating cortisol is a major factor that alters the activity of GRs, thereby changing the functions of GRs in the hippocampus, ultimately leading to a situation where the activity of the ACTH and CRH cannot be controlled; this seems to be responsible for depressive symptoms [3]. The HPA axis is a liable factor for increased circulating GC-mediated stress response; thus, high GC levels are a signal for negative feedback mechanisms to turn off the HPA activity in the hippocampus.

Previous studies have reported that chronic stress downregulates the hippocampal GR expression; GRs play a critical role in affecting hippocampal function [3, 4]. Excessive circulating GC levels are associated with not only the loss of GR-containing cells in the paraventricular nucleus but also decreased GR gene expression [5, 6]. It is hypothesized that the application of different stressors and the specific durations of the stress may cause alterations in the GR expression in certain rodent strains [7]. Furthermore, previous studies have revealed that the levels of neuronal nitric oxide synthase (nNOS), an endogenous inhibitor of GR in the hippocampus, increase upon exposure to stress [8] and are involved in the regulation of the HPA axis activity via GRs [9]. Thus, the inhibition of nNOS in the hippocampus leads to the decrease of the CORT concentration in the plasma and reduced CRH expression in the hypothalamus. Growing evidence has shown that a persistent increase in the circulating GC levels negatively affects neurogenesis [10]. In subjects with depression, the expression of brain-derived neurotrophic factor (BDNF), which is required for neurogenesis, eventually decreases; this is associated with high GC levels, and signaling for the promotion of neurogenesis, including the phosphorylation of the signaling molecules cAMP response element-binding protein (CREB) and extracellular regulated kinase (ERK), in the hippocampus is altered, which may negatively affect the HPA axis activity. Presently, an increasing number of herbal medicines with potent antidepressant effects have become the major focus of attention to treat depressive moods, as these natural herbs, which may include plants such as *Piper methysticum* and St John's wort, are associated with a higher level of safety [11].

We have been reported that *So-ochim-tang-gamibang* (SOCG) is a therapeutic agent for stress-related disorders such as depression [12]. SOCG is based on So-ochim-tang, which had described in the qi chapter of Donguibogam, a Korean traditional medical book [13]. After reformulation by replacing Aquilariae Resinatum Lignum to Aucklandia Radix (*Aucklandia lappa* DC.) and adding Aurantii Fructus (*Citrus aurantium* L.) and Platycodi Radix (*Platycodon grandiflorus* Jacq. A.DC) according to Bang-yak-hap-pyeon [14], SOCG has been prescribed for treating mental activity- and depression-associated somatoform pain [15]. We have reported that SOCG has therapeutic effects against stress-induced depression and is safe for use [16, 17]; the antidepressive effects of SOCG may be mediated *via* its neuroprotective activity and ability to lower the circulating GC concentration [12]. However, the mechanisms underlying the SOCG-mediated regulation of HPA hyperactivity have not yet been investigated at a molecular level. Wistar Kyoto (WKY) rats represent a convenient rodent model of depression; in these rats, depressive episodes can be reversed by antidepressants [18]. By utilizing *in vitro* bioassays and *in vivo* models, the effects of SOCG on the hyperactivity of the HPA axis were evaluated with a focus on stress hormone regulation, GRs, and nNOS, as well as the BDNF/CREB/ERK signaling pathways.

## 2. Materials and Methods

### 2.1. Animals

Male Wistar Kyoto rats (10 weeks old, 180–200 g) were provided by Dr. Tae Wan Kim from the Kyungpook National University, Daegu, the Republic of Korea, for our experiments. The animals were maintained at standard conditions (12 : 12 h light-dark cycle, 23 ± 1°C), with free access to water and food. The rats were subjected to one week of acclimatization before the experiments were begun. All the experiments involving animals were carried out in accordance with the National Institute of Health Guide for the Care and Use of Laboratory Animals (NIH publication no. 85-23, revised 1985), and the Institutional Animal Care and Use Committee of the Daejeon University approved the experimental protocol (DJUARB2015-030).

### 2.2. Experimental Design and Drug Administration

The preparation of SOCG and the choice of its standard dose (300 mg/kg) were based on a recently published report [12]. Briefly, Cyperi Rhizoma (*Cyperus rotundus* L.), Lindera Radix (*Lindera strychnifolia* Fern.-Vill.), Aucklandiae Radix (*Aucklandia lappa* Decne.), Glycyrrhizae Radix (*Glycyrrhiza uralensis* Fisch.), Aurantii Fructus (*Citrus aurantium* L.), and Platycodi Radix (*Platycodon grandiflorus* Jacq. A.DC) (8 :  4 :  1 :  1 :  4 :  4, in the order given) were boiled together in distilled water at 100°C for 2 h. The extract was evaporated and freeze-dried. The SOCG powder was stored at −20°C. The drug-extract ratio (DER) was 6.8 (yield ratio, 14.6%). Voucher specimens (no. 194A079-85) were deposited in the herbarium of Han Kook Shin Yak Co., Ltd. (Nonsan, Korea). Naive rats were housed under identical conditions in a separate room and had no contact with the animals in the stressed (control) and treatment groups. The rats in the chronic restraint stress group were placed in acrylic cylinders (250 mm long × 75 mm diameter, with air vents at the nasal end of the cylinder) for 3 h (09:00 to 12:00 h) every day from the age of 8 or 9 weeks for 2 weeks. The rats were assigned to four experimental groups: (a) naive, no restraint stress (*n* = 6); (b) con, restraint stress with vehicle (*n* = 6, saline, 0.9% NaCl); (c) AMI, restraint stress with 10 mg/kg amitriptyline (*n* = 5, per oral administration, Sigma-Aldrich Co., St. Louis, MO, USA); and (d) SOCG, restraint stress with 300 mg/kg SOCG (*n* = 6, per oral administration).

### 2.3. Depression-Like Behavior Tests

After the repeated administration of the restraint stress, the animals were subjected to the open field test (OFT), forced swimming test (FST), and sucrose preference test (SPT). All tests were performed between 09:00 and 14:00 h in a quiet room.

The locomotion behavior of rats was assessed via the OFT. The open-field arena (100 × 100 × 50 cm) was constructed using acrylic sheets. Their behavior was observed and videotaped for 10 min using a video tracking software (SMART 3.0; Panlab SI, Barcelona, Spain). Travel distance (*m*) was used as the parameter to measure locomotor activity.

In the FST, the rats were individually forced to swim in a cylinder (40 cm high × 18 cm diameter) containing tap water (25 ± 2°C), and the cylinder was long enough to not let the animals escape or rest by touching the bottom. The rats were forced to swim for 15 min (pretest) on the day before the session. After 24 h of the pretest, each session lasted 360 sec, and the duration of immobility was recorded. Mobility is defined as any movement beyond what is necessary to maintain the head above water. The total immobility time was measured during the last 4 min of each session using video tracking software (SMART 3.0; Panlab SI, Barcelona, Spain). The first 2 min of each session is acclimated time for immobility.

In the case of the SPT, prior to the testing, the animals were trained to adapt to a sucrose solution (1%, w/v); two bottles of sucrose solution were placed in each cage for 24 h, and one bottle of sucrose solution was then replaced with water for 24 h. After the adaptation, the rats were deprived of water and food for 24 h. During the test, the rats were housed in individual cages for 3 h and had free access to both sucrose solution and water. After this time period, the water and sucrose bottles were removed, and the consumed volumes were noted. Sucrose preference was calculated from the data obtained after 3 h of testing as follows: (volume of sucrose solution consumed/total volume of liquid consumed) × 100%.

### 2.4. Analysis of Plasma Stress Hormone Levels

One day after the behavioral tests, the animals were anesthetized via the inhalation of laboratory ether; the blood from the rats was collected into Vacutainer tubes containing EDTA (Becton Dickinson, NJ, USA) by heart puncture. The plasma was isolated by the centrifugation of the blood samples for 15 min at 3,000 rpm; the supernatants were collected. The plasma was stored at −80°C until further analysis. The plasma levels of CORT (Enzo Life Sciences, NY, USA), ACTH (Cloud-Clone Corp., TX, USA), and CRH (Cloud-Clone Corp., TX, USA) were measured using commercially available ELISA kits, according to the manufacturer's protocols.

### 2.5. SH-SY5Y Cell Culture

The neuroblastoma cell line SH-SY5Y (American Type Culture Collection, VA, USA) was maintained in DMEM supplemented with 10% heat-inactivated FBS and 1% (v/v) penicillin/streptomycin at 37°C in an atmosphere of 5% CO_2_ and 95% air. The medium was replaced every 2–3 days.

### 2.6. Cell Viability Assay

The cells were seeded into a 96-well plate at a density of 2 × 10^5^ cells/well and incubated for 24  h; then, they were exposed to various doses of SOCG (1, 10, 100, or 500 *μ*g/ml) or CORT (100, 200, 300, and 400 *μ*M) for another 24  h. To evaluate the protective effects of SOCG against CORT-induced cell injury, the cells were coincubated with SOCG (1, 10, 100, or 500 *μ*g/ml) for 2 h and then with CORT (100 *μ*M) for another 24 h. At the end of the treatment process, 10 *μ*l of MTT (EZ cytox, DoGenBio Co., Ltd., Seoul, South Korea) was added to each well, followed by incubation for 4 h. The absorbance of the samples was measured at 450 nm using a microplate reader (Molecular Device, Sunnyvale, CA, USA). Cell viability was expressed as the percentage of viable cells relative to the nontreated control.

### 2.7. Western Blotting

After the whole brains were isolated, the hippocampus, hypothalamus, and pituitary gland were immediately dissected while the brains were placed on an ice surface. The tissues were stored at −80°C until further use. The SH-SY5Y cells were seeded into a six-well culture plate at a density of 2 × 10^5^ cells/well for 24 h; they were pretreated with SOCG (1, 10, or 100 *μ*g/ml) for 1 h and then treated with CORT (100 *μ*M) for 24 h. They were then rinsed with ice-cold PBS and lysed in the RIPA buffer. Equal amounts of each protein sample were resolved on 8–18% sodium dodecyl sulfate-polyacrylamide gels; the resultant bands were transferred onto nitrocellulose membranes (Hybond ECL; Amersham Pharmacia Biotech, Piscataway, NJ, USA). The membranes were blocked in 5% skim milk solution for 1 h. Next, they were incubated with antibodies against BDNF, CREB, GR (1 : 1000; Santa Cruz Biotechnology Inc., CA, USA), ERK (1 : 1000; Cell Signaling Technology, MA, USA), and nNOS (1 : 1000; Merck, Darmstadt, Germany) overnight at 4°C and then with horseradish peroxidase-labeled IgG antibodies (1 : 2000; Santa Cruz Biotechnology Inc., CA, USA) for 2 h at room temperature. For the detection of the protein bands, the ECL Western Blotting Detection System (Amersham Biosciences, PA, USA) was used. The band intensities were measured using the ImageJ software (version 1.49).

### 2.8. Real-Time PCR

SH-SY5Y cells were seeded in a 6-well plate at a density of 2 × 10^5^ cells/well. The cells were pretreated with SOCG for 1 h, followed by incubation with CORT for 24 h to measure the mRNA expression levels. Total RNA was isolated using the TRIzol reagent (Invitrogen) and then used for cDNA synthesis, which was performed with the PrimeScript™ RT reagent kit (TaKaRa, Shiga, Japan). The specific genes were quantified using a 7500 Real-Time PCR System (Applied Biosystems, CA, USA), with the Power SYBR® Green PCR Master Mix and TaqMan® Gene Expression Master Mix (Applied Biosystems, CA, USA). The sequences of the real-time PCR primers used were as follows: rat BDNF, forward 5'-CAGCTGGGTAGGCCAAGTTG-3' and reverse 5'-CACAATGTTCCACCAGGTGAGA-3', and rat GR, forward 5'-GGCTGAGCAGATTACATAGGC-3' and reverse: 5'-GATGGAAAGGGGCCTTTTGG-3'. A rat GAPDH primer set (Endogenous Control, VIC®/MGB Probe, Primer Limited) was purchased. For the PCR analysis of the SH-SY5Y cells, the sequences of the primers used for the real-time PCR analysis included the following: human BDNF, forward 5'-CCAACGGATTTGTCCGAGGT-3' and reverse 5'-ATCTCAGTGTGAGCCGAACC-3'; human GR, forward 5'-GGACCACCTCCCAAACTCTG-3' and reverse 5'-GCTGTCCTTCCACTGCTCTT-3'; and human GAPDH (Endogenous Control), forward 5'-TGAAGACGGGCGGAGAGAAAC-3' and reverse 5'-TGACTCCGACCTTCACCTTCC-3'. The PCR was run for 40 cycles at 95°C (15 sec) and 60°C (1 min). The relative expression levels of the target genes were calculated using the ΔΔCt method, where Ct is the threshold concentration.

### 2.9. Statistical Analysis

The data were analyzed using GraphPad Prism 5 and represented as the mean ± SEM in all experiments, except in the MTT test, in which they were represented as the mean ± SD. Comparisons between the data from different groups were performed using one-way ANOVA followed by Tukey's or post hoc analyses. *P* values < 0.05 were considered statistically significant [19].

## 3. Results

### 3.1. SOCG Decreased Depressive-Like Behaviors in WKY Rats

**Figure 1:**
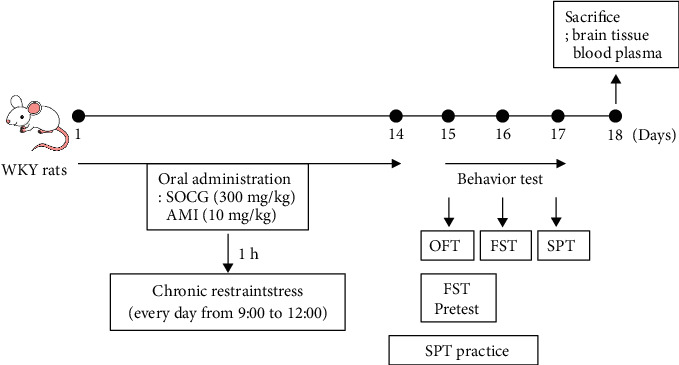
Schematic diagram of the animal experimental procedure. WKY: Wistar Kyoto, SOCG: *So-ochim-tang-gamibang*, AMI: amitriptyline, OFT: open field test, FST: forced swimming test, and SPT: sucrose preference test.

**Figure 2:**
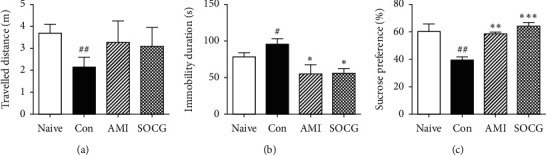
Effects of SOCG on depressive-like behavior in WKY rats. The animals were subjected to chronic restraint stress for 2 weeks, and SOCG was orally administered (300 mg/kg). (a) Travelled distance in the OFT, (b) immobility duration in the FST, and (c) sucrose preference in the SPT were measured. The data are presented as the mean ± SEM. ##*P* < 0.01, significant difference compared to the naive group. *P* < 0.05, ^*∗∗*^*P* < 0.01, and ^*∗∗∗*^*P* < 0.001, significant difference compared to the control group. AMI: amitriptyline (10 mg/kg).

First, we evaluated the antidepressive effects of SOCG in WKY rats by performing behavioral tests including OFT, FST, and SPT (Figure 1). In the OFT, the control rats showed reduced locomotion activity (2.15 ± 0.44 m, *F*[3, 19] = 0.911, *P* < 0.01) compared to the naive rats (3.69 ± 0.41 m). The AMI- and SOCG-treated rats showed an increased locomotion distance (3.27 ± 0.98 m and 3.08 ± 0.87 m, respectively) compared to the control rats (Figure 2(a)). In the FST, the control rats exhibited an increased immobility duration (94.84 ± 7.60 s, *F*[3, 19] = 5.876, *P* < 0.05) than the naive rats, while SOCG significantly suppressed the immobility duration time of the rats (54.67 ± 6.41 s, *P* < 0.05), compared to the case for the control rats (Figure 2(b)). Next, in the SPT, we found that the sucrose consumption was reduced in the case of the control rats (39.06 ± 2.33%, *F*[3, 19] = 9.393, *P* < 0.01) compared to the naive rats (60.39 ± 5.54%). However, SOCG treatment increased the sucrose preference in the restraint stress-treated WKY rats (64.39 ± 2.79%, *P* < 0.05), compared to the case for the control rats (Figure 2(c)). These results indicate that SOCG alleviated depressive-like behaviors in WKY rats subjected to restraint stress. SOCG ameliorated the circulating stress hormone levels.

### 3.2. SOCG Ameliorated the Circulating Stress Hormone Levels

**Figure 3:**
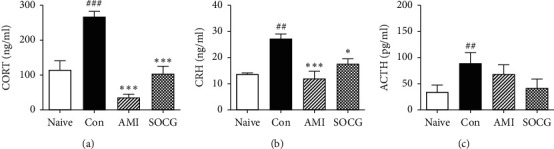
Effects of SOCG on the plasma levels of HPA axis hormones. WKY rats were subjected to chronic restraint stress for 2 weeks, and SOCG was orally administered (300 mg/kg). The plasma levels of (a) corticosterone, (b) corticosterone-releasing hormone, and (c) ACTH were measured by ELISA. The data are presented as the mean ± SEM. ##*P* < 0.01 and ###*P* < 0.001, significant difference compared to the naive group. ^*∗*^*P* < 0.05 and ^*∗∗∗*^*P* < 0.001, significant difference compared to the control group. AMI: amitriptyline (10 mg/kg).

Since the HPA axis is strongly activated under conditions of uncontrollable stress, we investigated the plasma levels of the HPA axis hormones CORT, CRH, and ACTH by ELISA. The plasma levels of CORT (266.6 ± 15.72 ng/ml, *P* < 0.001), CRH (26.75 ± 1.95 ng/ml, *P* < 0.01), and ACTH (85.35 ± 21.76 pg/ml, *P* < 0.01) were significantly higher in the restraint stress-treated rats than in the naive rats (113.50 ± 27.41 ng/ml, 12.84 ± 0.60 ng/ml, and 28.91 ± 13.94 pg/ml, Figures 3(a)–3(c)). In contrast, SOCG treatment ameliorated the plasma CORT (102.30 ± 23.07 ng/ml, *P* < 0.001), CRH (16.85 ± 2.12 ng/ml, *P* < 0.05), and ACTH (36.90 ± 18.10 pg/ml) levels, compared to the case for the control rats. Collectively, these data indicate that SOCG treatment ameliorated the chronic stress-induced increase of the levels of circulating HPA axis hormones in a rat model of depression. SOCG downregulated the ACTH and CRH levels in the pituitary gland and hypothalamus, respectively.

### 3.3. SOCG Downregulated the ACTH and CRH Levels in the Pituitary Gland and Hypothalamus

**Figure 4:**
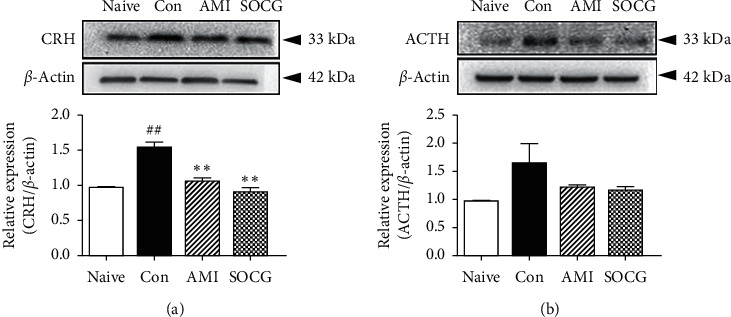
Effects of SOCG on the levels of HPA axis hormones in the brain. WKY rats were subjected to chronic restraint stress for 2 weeks, and SOCG was orally administered (300 mg/kg). The pituitary gland and hypothalamus were isolated from the rat brains and the levels of (a) CRH and (b) ACTH, respectively, were measured by western blotting. The data are presented as the mean ± SEM. ##*P* < 0.01, significant difference compared to the naive group. ^*∗∗*^*P* < 0.01, significant difference compared to the control group. AMI: amitriptyline (10 mg/kg).

Next, we investigated the levels of ACTH and CRH in the pituitary gland and hypothalamus, respectively, where the HPA axis hormones are produced. The protein levels of CRH in the hypothalamus increased following restraint stress; however, SOCG significantly reduced the CRH concentration (by approximately 60%) compared to the case for the samples from the control rats (Figure 4(a)). SOCG treatment downregulated the restraint stress-induced ACTH overexpression in the pituitary gland (Figure 4(b)). These results suggest that SOCG acted on specific sites in the brain to inhibit the excess production of HPA axis hormones.

### 3.4. Effects of SOCG on the Hippocampal Expression of HPA Axis-Related Signaling Molecules

**Figure 5:**
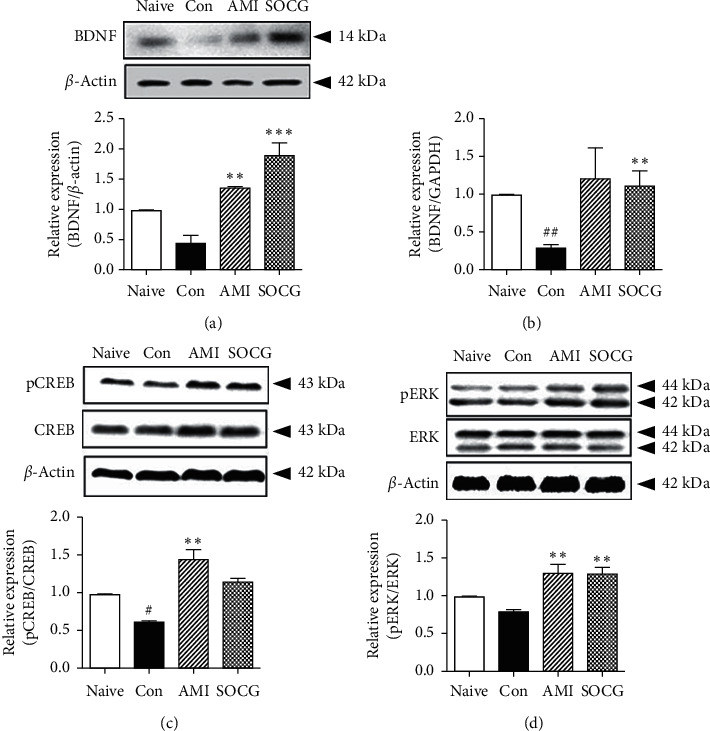
Effects of SOCG on the expression of neuronal factors in the hippocampus. WKY rats were subjected to chronic restraint stress for 2 weeks, and SOCG was orally administered (300 mg/kg). The hippocampus was dissected from the rat brains and used for protein and mRNA purification. The levels of BDNF were measured by (a) western blot analysis and (b) real-time PCR. The levels of (c) phosphorylated CREB and (d) phosphorylated ERK were measured by western blotting analysis. The data are presented as the mean ± SEM. #*P* < 0.05 and ##*P* < 0.005, significant difference compared to the naive group. ^*∗∗*^*P* < 0.01 and ^*∗∗∗*^*P* < 0.001, significant difference compared to the control group. AMI: amitriptyline (10 mg/kg).

We examined the hippocampal BDNF expression, which is controlled by HPA axis hormones, by western blotting analysis. SOCG strongly increased the BDNF levels in the hippocampus (Figure 5(a)). The expression of BNDF at the RNA level was confirmed by real-time PCR. SOCG treatment significantly reversed the decrease in the hippocampal BDNF mRNA levels in the stressed animals (Figure 5(b)). We also investigated the activation of CREB and ERK, which regulate the expression of BDNF following the stimuli of the HPA axis hormones. There were notable decreases in the levels of phosphorylated CREB (Figure 5(c)) and ERK (Figure 5(d)) in the control rats, compared to the case for the naive rats. However, SOCG treatment increased the levels of CREB and ERK phosphorylation (Figures 5(c) and 5(d)). These results suggest that SOCG improved depression-like behaviors *via* regulating the expression of HPA axis-related signaling molecules.

### 3.5. Effects of SOCG on the Hippocampal GR Expression

**Figure 6:**
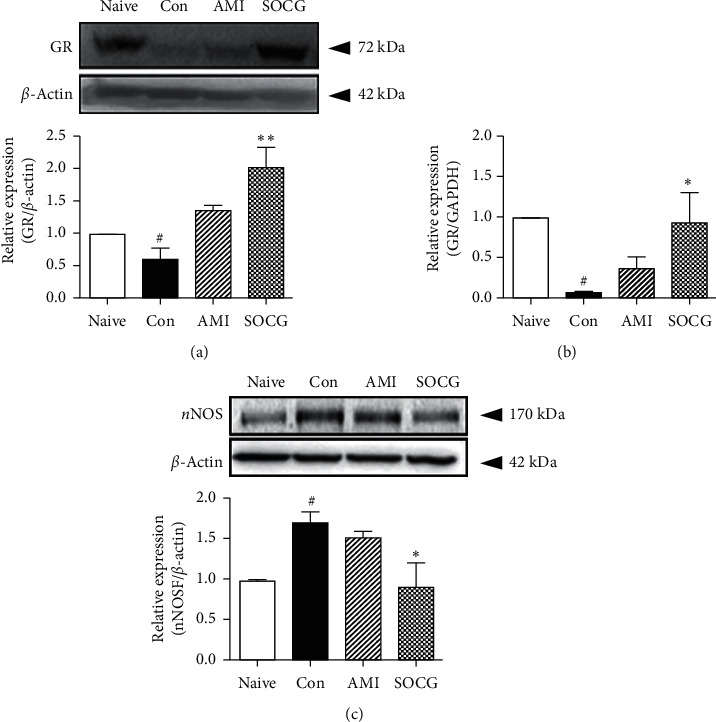
Effects of SOCG on the hippocampal GR and nNOS expression levels in WKY rats. The animals were subjected to chronic restraint stress for 2 weeks, and SOCG was orally administered (300 mg/kg). The hippocampal GR levels were measured by (a) western blotting analysis and (b) real-time PCR. (c) The hippocampal nNOS levels were measured by western blotting analysis. The data are presented as the mean ± SEM. GAPDH was used for the normalization of the expression levels of the target genes in the real-time PCR analysis. #*P* < 0.001, significant difference compared to the naive group. ^*∗*^*P* < 0.05 and ^*∗∗*^*P* < 0.01, significant difference compared to the control group. AMI: amitriptyline (10 mg/kg).

The GR, a receptor of CORT, mediates the activity of the GC hormone [19]. The GR level in the hippocampus increased significantly following SOCG treatment (by approximately 3-fold), compared to the case for the samples from the control rats, which showed lower GR levels than those from the naive rats, as revealed by the western blotting analysis (Figure 6(a)). Moreover, SOCG dramatically increased the mRNA expression levels of GR in the hippocampus, compared to the case for the samples from the control rats (Figure 6(b)). nNOS is known to regulate the activity of the HPA axis; it inhibits the activity of GRs in the hippocampus [20]. Thus, we measured the hippocampal nNOS expression in the depressed animals. Restraint stress increased the hippocampal nNOS levels in samples from the depressed rats, compared to the case for those from the naive rats. Notably, SOCG significantly suppressed the hippocampal nNOS expression to a level similar to that observed in the samples from the naive rats (Figure 6(c)). Thus, SOCG may control the hyperactivity of the HPA axis *via* the upregulation of hippocampal GR expression and inhibition of nNOS expression in depressed animals.

### 3.6. Effects of SOCG on the Expression of HPA Axis-Related Neuronal Factors in SH-SY5Y Cells

**Figure 7:**
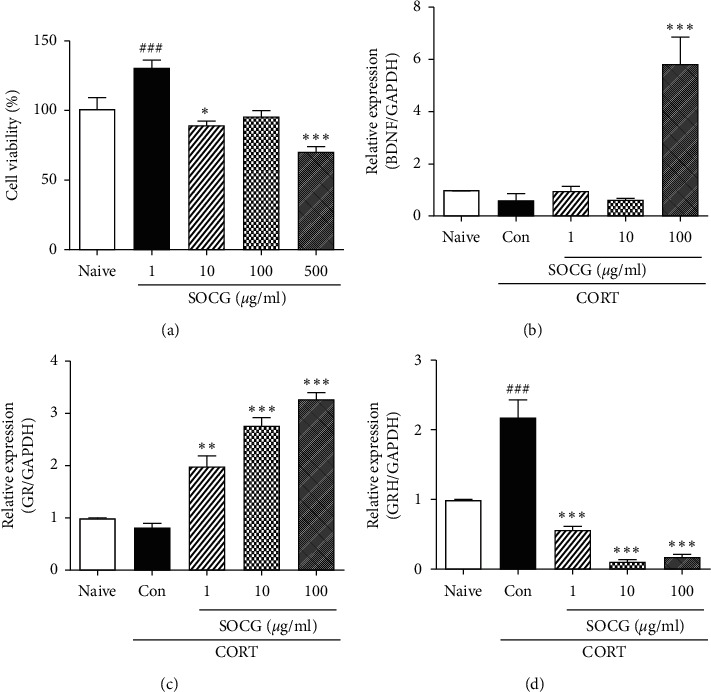
Effects of SOCG on the expression of HPA axis-related molecules in SH-SY5Y cells. (a) The viability of SH-SY5Y cells was determined by the MTT assay after they were cocultured with various concentrations of SOCG for 24  h. The cells were pretreated with SOCG for 1 h followed by incubation with corticosterone (100 *μ*M) for 24 h for measuring the (b) BDNF, (c) GR, and (d) CRH mRNA expression levels. Total RNA was isolated and the levels of genes were measured by real-time PCR. The data are presented as the mean ± SEM. GAPDH was used for the normalization of the expression levels of the target genes in the real-time PCR analysis. ###*P* < 0.001, significant difference compared to the naive group. ^*∗*^*P* < 0.05, ^*∗∗*^*P* < 0.01, and ^*∗∗∗*^*P* < 0.001, significant difference compared to the control group. CORT: corticosterone.

We confirmed the effects of SOCG on the HPA axis *in vitro*. First, we tested the cytotoxicity of SOCG in cells from a neuronal cell line, i.e., SH-SY5Y cells. Treatment with SOCG (1, 10, and 100 *μ*g/ml) for 24 h did not show cytotoxicity, except in case of treatment with 500 *μ*g/ml (*P* < 0.001) SOCG (Figure 7(a)). Therefore, we used SOCG concentrations of less than 100 *μ*g/ml for the following tests. The BDNF expression in CORT-treated SH-SY5Y cells was assessed by real-time PCR. CORT treatment decreased the BDNF mRNA expression level; however, it strongly increased the BDNF expression (by more than 6-fold), compared to the samples from the control rats (Figure 7(b)). SOCG also increased the GR expression in a dose-dependent manner in CORT-treated SH-SY5Y cells (Figure 7(c)). On the other hand, SOCG downregulated the CORT-induced increase of the CRH mRNA expression levels in the SH-SY5Y cells (Figure 7(d)). These *in vitro* results confirmed the antidepressive effects of SOCG *via* its action on the HPA axis.

## 4. Discussion

It is well known that exposure to chronic stress affects HPA axis activity, thereby increasing the synthesis and secretion of GCs. The HPA axis is one of the major endocrinological systems that control stress. The increase in the circulatory levels of GC is the human body's response to stress. However, when the stress is persistent or uncontrollable, the HPA axis activity is abnormally regulated, resulting in depression [2]. Therefore, proper response to stress-induced alterations in the activity of the HPA axis can be a therapeutic target for depression. Our previous study has proposed the hypothesis that SOCG may control the hyperactivity of the HPA axis since SOCG downregulated the plasma levels of CORT, which is a major HPA axis-related stress-responding hormone, in a mouse model of stress-induced depression [12]. In this study, we showed that SOCG administration inhibited the increase in the plasma levels of stress-related HPA axis hormones including CORT, CRH, and ACTH. Since the hypothalamic CRH and pituitary ACTH levels were also downregulated by SOCG, it seems that SOCG controls the secretion of CRH and ACTH in the central nervous system. Our data strongly evidence that SOCG ameliorates depression-related symptoms by controlling the expression of HPA axis hormones.

The therapeutic effects of SOCG in the hippocampus appear to be elicited by its ability to increase the expression of neurotrophins *via* the control of the HPA axis activity. In accordance with our previous study using a mouse model, the BDNF expression, which was downregulated in the hippocampus of depressed rats, increased following SOCG treatment. Since the hippocampal level of BDNF is closely correlated with the severity of depressive symptoms, the increased expression of neurotrophins in the brain reflects the antidepressive effects of SOCG [21]. Further, our data suggest that SOCG increased the BDNF expression by modulating the phosphorylation of CREB and ERK. CREB is a transcription factor regulating the expression of neurotrophins such as BDNF, which is controlled by the activity of ERK [22, 23]. The activation of ERK signaling cascades, along with CREB signaling cascades, mediates neuronal activities such as neuronal cell differentiation, survival, and synaptic plasticity [24]. As BDNF plays a critical role in the therapeutic process of depression, the increase in the hippocampal BDNF levels may be an important therapeutic molecular mechanism underlying the antidepressant activity of SOCG [25]. The secretion of CORT in response to stress activates c-fos in the brain, leading to the suppression of the BDNF expression in the brain [26]. This suggests that the HPA axis is closely related to the BDNF expression. Our data demonstrated that SOCG suppressed the CORT levels in the blood but elevated the BDNF expression. The increase of the hippocampal BDNF levels may result from the SOCG-mediated modulation of the HPA axis activity. However, the detailed molecular mechanisms underlying the SOCG-mediated control of BDNF expression *via* the HPA axis remain to be elucidated.

It has been suggested that appropriate signaling by the GRs plays a critical role in the regulation of the HPA axis in the brain. Especially, hippocampal GR activation is important for GC-mediated negative feedback action of the HPA axis to control CORT release and hormonal homeostasis [27]. Therefore, it has been suggested that GR may be a target of antidepressants [28]. Our studies examining the consequences of chronic stress revealed that the GR levels in the hippocampus were downregulated [4]. With respect to chronic stress, the decreased GR level reflects the insensitivity of CORT stimulation in the brain, which indicates a broken HPA axis circuit. However, SOCG was able to restore the GR levels in a rat model of depression. This may induce the recovery of the sensitivity of CORT and the activity of the HPA axis, resulting in the antidepressive effects of SOCG. Although the detailed molecular mechanisms underlying these phenomena must be investigated, the SOCG-mediated increase in the GR levels may contribute to the restoration of depressed rats.

It is well known that higher nNOS expression in the hippocampus leads to neuronal loss under chronic stress conditions. Our study revealed that nNOS was overexpressed in the hippocampus in depressed animals, while SOCG reversed this increase of nNOS expression [29]. This downregulation of hippocampal nNOS expression by SOCG is quite interesting because suppressed NO production in the hippocampus may promote hippocampal neurogenesis in patients with depression [30]. Therefore, SOCG may improve depression symptoms by modulating the NO production.

SOCG is formulated by composing 6 herbs. Some active compounds from each herb showing antidepressive effects were reported. Cycloartane and iridoid isolated from *Cyperus rotundus* were demonstrated to have beneficial effects on depression by animal experiments with FST and TST [31]. Glutamate-induced neuronal cell death was protected by platycodin from *Platycodon grandiflorum* [32]. Antidepressive effects of liquiritin from *Glycyrrhiza uralensis* were investigated in the chronic stress-induced depression rat model by SPT and FST [33]. Essential oil from *Citrus aurantium*, which contains 97.83% of limonene and 1.43% of myrcene, was reported to have anxiolytic effect [34]. Those compounds would play roles in antidepressive effects of SOCG, and there is a possibility that they may act synergistically; however, identification of the active compound of SOCG needs to be investigated.

## 5. Conclusion

In conclusion, SOCG controlled the levels of HPA axis hormones in a rat model of chronic stress-induced depression. SOCG suppressed the stress-induced increase of the circulating plasma levels of the HPA axis hormones, decreased the ACTH and CRH levels in the pituitary and hypothalamus, respectively, and increased the hippocampal expression of GR, which is an important receptor for the GC hormone. In SOCG-treated depressed rats, an upregulation of the hippocampal expression of the HPA axis-related signaling molecules CREB and ERK was observed; this may lead to the increase of BDNF expression. Furthermore, SOCG treatment increased the GR and BNDF mRNA expression levels and ameliorated the CORT-induced increase in the CRH mRNA expression levels in CORT-treated SH-SY5Y cells. The effects of SOCG on the expressions of CRH and GR were confirmed in SH-SY5Y cells, human neuroblastoma cells. SH-SY5Y cells are widely used as an *in vitro* model of neuronal diseases including depression, Alzheimer's disease, and Parkinson's disease. As the characteristics of the cells are not the same as that of CRH-releasing neurons in the hypothalamus, a further *in vitro* study is needed to verify the function of SOCG on the hypothalamus. Nevertheless, our *in vivo* and *in vitro* studies indicated that SOCG ameliorates depression-related symptoms by regulating the HPA axis. Further studies are warranted to explore the detailed molecular mechanisms underlying the endocrinological action of SOCG, which may serve as a promising antidepressive therapeutic agent.

## Data Availability

The datasets used and/or analyzed in the current study are available from the corresponding author upon reasonable request.
